# On the Use of Sulphate of Quinine in Typhoid Fever

**Published:** 1846-05

**Authors:** A. N. Bell

**Affiliations:** Waterbury, Ct.


### *On the Use of Sulphate of Quinine in Typhoid Fever.* By A. N. Bell, M. D.

Since about the first of September last, typhoid fever has been prevalent in Waterbury, (Ct.,) and from its general fatality under the usual mode of treatment, and my knowledge of the effects of sulphate of quinine in other fevers, I adopted the following practice, since which time I have not lost a patient with it.

Case I.—A. A., a laboring man, æt. 26, of strong constitution, having always enjoyed good health, and for the last two years lived an intemperate life, exposing himself to damp weather, night air, and the usual concomitants of such a life, sent for me.

Sept. 22d. He has had pain in the head, back and limbs; very unrefreshing sleep and loss of appetite for a week; and, for the last two days, chilliness with shivering, followed by heat and partial perspiration. Has had three thin, alvine evacuations,—rather darker coloured than natural—during the last twenty-four hours: had none previously for two days. Has been troubled twice during the same period with epistaxis. He is now perspiring about his forehead and temples, but complains of chilliness as soon as the clothes are in the least removed. Has a small, frequent pulse,—98. The tongue is white and moist; there is but little thirst; no appetite; severe pain in the back; headache; soreness over the lower part of the abdomen, mostly in the left hypochondrium. The spleen can be felt slightly enlarged.

Prescription. ℞. Hydrarg. chlorid. mitis. ℈j.

Pulv. opii, grs. xij.

Ft. pulv. div. in chartul., iv.

S. One to be taken every six hours.

Diet. Toast water, or very thin Indian meal gruel.

Sept. 23. Expresses himself as having rested better, but to his attendants he seemed very much disturbed in sleep; talked almost incessantly about his business affairs. He seemed to be lost upon waking; started to get up; forgot he was sick. There is difficulty of attention, (very much so at this time.) Has had two alvine evacuations, very fœtid and dark in colour. The skin is drier and hotter; the pulse 106, quick and small; the tongue is drier and more thickly furred, white; there is more thirst; no pain in the head or back, only when he moves; tenderness of the abdomen is more confined to the left hypochondrium. There is no chilliness nor shivering.

Prescription. ℞. Hydrarg. chlorid. mitis.
Pulv. ipecac. et opii, *aa* gr. x. M.

To be taken at 6 o'clock, P. M. Diet the same; cold water if desired.

24th. His brow is contracted, and he is obstinate; does not wish to obey directions. Was delirious during the night. Has had no evacuation from the bowels. The skin is dry and hot, was a little moist for three hours after taking the powder. The pulse is 112, quick and fuller. The tongue is dry, its coat is darker; no pain in the back. There is pain in the head through the temples; more tenderness generally of the abdomen, less in the left hypochondrium.

Prescription. ℞. Hydrarg. chlorid. mitis.
Ext. colocynth. compos. *aa* gr. v. M.

To be taken now, (10 o'clock, A. M.)

25th. Is now in quiet sleep; upon waking seems lost. The eyes are vacant in expression; the brow is more contracted; he is drowsy; the attention cannot be confined; the skin is very hot and dry; pulse 120, quick, and smaller than yesterday; tongue more dry and darker coloured, cracked, glossy at the edges and swollen; thirst increased. The spleen can be felt,—no more enlarged; it is less tender. He was delirious during the night; wanted to get up frequently. Has had three darker coloured and more fœtid evacuations from the bowels. Epistaxis. Prescription. A tablespoonful of castor oil now, (11¼ o'clock, A.M.,) and a blister plaster, 5 × 7 inches, over the stomach, in case the sickness increases, or he has tenderness of the abdomen.

26th. The countenance is better; there is less drowsiness and more attention. The skin is not so hot, but it is dry; the pulse is 118, still quick and fuller; the tongue rather darker, otherwise the same; thirst the same. The blister plaster was applied at 7 o'clock, P. M., remained on 10 hours, drew well. He has been less delirious and drowsy; has headache, wandering pains about the abdomen; has had four alvine evacuations; appearances the same.

Prescription. ℞. Quiniæ sulphatis, ʒj.

Acid. sulphuric. aromat., fʒss.

Aquæ, f℥vi. M.

S. A tablespoonful to be taken every two hours.

27th. Symptoms are much as they were on the 25th et seq., excepting that the tongue is blacker, and more sordes. The pulse is 116. Sudamina. Has had one alvine evacuation. Prescription same as yesterday.

Oct. 8th. Up to this time the last prescription has been continued. He has taken no purgative medicine whatever, having had two or three alvine evacuations daily, until within the last twenty-four hours, during which he has had none. He is now very stupid; has thick sordes about his teeth and lips; the tongue is protruded with difficulty, and left so by his not being able to withdraw it; it is black, cracked, and bleeds. Pulse 120. Subsultus tendinum; slight meteorism.

Prescription. ℞. Quiniæ sulphatis, ʒj.

Piperin. gr. xij.

Acid. sulphuric. aromat. gtt. xxx.

Aquæ, f℥vj. M.

S. A tablespoonful to be taken every hour.

Called at 7 o'clock, P. M. Has hæmatemesis. Directed a lump of ice to be held in the mouth, but which he swallowed, and it was soon effectual. Had an evacuation from the bowels about an hour ago, very dark coloured and excessively fetid. Discontinued the prescription of this morning.

Prescription. ℞. Quiniæ sulphatis, ʒj.

Syr. acaciæ q. s.

M. f. pil. xxiv.

S. Two to be taken every hour.

9th. Appears much the same as yesterday; meteorism does not seem to have increased any; has had four alvine evacuations; pulse 110. Prescription continued.

13th. Up to this time from the 9th, he has had very much the same symptoms. The bowels have been open on an average of from four to six hours daily. He has had hæmatemesis again, and it was controlled by the same means as before. The last prescription has been continued. Pulse 118.

Prescription. ℞. Quiniæ sulphatis, ʒij.

Piperin. gr. xij.

Acid. sulphuric. aromat. fʒss.

Aquæ, f℥vi.

M. S. A tablespoonful to be taken every hour.

14th. Seemed to rest better after midnight last night than for a week before. Works at his ears a great deal for the last few hours. Has had three alvine evacuations, much the same in appearance; there is no meteorism. The spleen cannot be felt, for the first time since it was first observed. He is less delirious, but complains very much of wagons constantly driving by; says he has something alive in his head. Pulse 100, not so quick; appearance of tongue and mouth the same.

Prescription. ℞. Quiniæ sulphatis, ℨss.

Acid. sulphuric. aromat., gtt. xx.

Aquæ, f℥vj.

M. S. A tablespoonful to be given every two hours.

19th. The same prescription has been continued. The alvine evacuations have gradually improved in appearance; the coat is separating from the tip and edges of his tongue, leaving it of natural colour and moist. Pulse 90, soft and slow; skin much more natural. Still complains some of his ears. The same medicine to be given once in four hours.

23d. He has continued to improve and is now decidedly convalescent. The last medicine is ordered three times a day.

Case II.—Miss E. P., æt. 21, sent for me this morning, Sept. 23d. Has enjoyed good health since she was twelve years old. About ten days ago she began to lose appetite, have pain in head, back and limbs, feel very tired, rest badly; had vertigo and drowsiness during the day, which she thought to be owing to close confinement and attention to her sick mother. On the 19th she was chilly most of the day; had increased pain in the head, back and limbs. Upon the going off of the chilliness she felt very hot, and tried to sweat it off by taking senna and salts, and warm drinks. The medicine produced three alvine evacuations; sweated her some, but she thought she felt much more debility on account of it in the morning. She has had perspiration about her forehead, temples and hands most of the time since, and has had an evacuation from the bowels daily. This morning, before sending for me, she took some aperient pills of her mother's; does not know their composition. They have operated once. Has pain in the stomach and right hypochondrium; feels great debility; pulse 112, hard and quick; tongue thickly coated, yellow; skin dry and hot. Prescription. Emplast. cantharides, 4+6 inches, to be applied over the stomach, inclining to the right side. Diet. Toast water, cold drinks and ice.

24th. Blister plaster has drawn well, but she still has some tenderness and pain in the right side. Has rested none during the night. The pills have operated four times. Pulse 120, quick and wiry; tongue darker coloured and dryer. There are sordes about the teeth. Sudamina. Snuffing respiration. "Feels a good deal worse," but can't describe it. Has much thirst; no disposition to speak, or to be spoken to.

Prescription. ℞. Quiniæ sulphatis, ʒj.

Morphiæ sulphatis, gr. ij.

Acid. sulphuric. aromat., fʒss.

Aquæ, ℥vj. M.

S. A tablespoonful to be taken every two hours. The blister to be dressed with savine cerate.

25th. Rested better last night, and feels better. Pulse 124, character the same; tongue dark brown and drier; more sordes; skin hotter and dry; headache; pain in the side and stomach continues; has had two dark coloured and fetid evacuations from the bowels. Prescription continued.

29th. Has continued the same prescription up to this time. The alvine evacuations have become more frequent; she has needed no apperient medicine. Pulse 140, quick, small and irregular; tongue black, rough and dry; has much thirst; no pain; has become tired of toast water, and gum arabic water acidulated with lemon, has been substituted. Medicine the same, to be taken every hour.

Oct. 5th. The sulphate of quinine has been continued up to the present time. The last prescription occasioned its peculiar influence on the head in less than twenty-four hours; and the dose was accordingly lessened, since which time she has been improving. When, on Sept. 30th, the effect of the quinine was first observed, her pulse was reduced in frequency, from 140 of the day before, to 116; and it has grown slower daily, until at this time it beats only 96 in the minute, and is soft, and perfectly regular. The tongue is beginning to clean.

Prescription. ℞. Quiniæ sulphatis, gr. xxiv.
Acid. sulphuric aromat., gtt. xx.
Aquæ, f℥vj. M.

S. A tablespoonful three times a day.

Case III.—G. N. P., æt. 23, carpenter, of good habits, and never sick before. Sent for me this morning, Nov. 9th. Has been confined to his bed two days, taking hot drinks for the purpose of trying to overcome the chilliness and shivering, which has troubled him for several days. But day before yesterday he felt so weak, had such a bad taste in his mouth, and having slept scarcely any for two or three nights, he concluded that he would lie by and doctor a little. Took some boneset tea, which vomited him very well, and has continued warm ginger tea up to this time. Is now in partial perspiration, wrapped up very warm and desires to remain so, because he is so cold otherwise. Has no appetite and a good deal of thirst. Has some griping pains in various parts of his abdomen, which he thinks are owing to the diarrhœa, which he supposes is all that is the matter with him, because that was his first trouble about a week ago, and still continues. Has had five or six operations from his bowels daily. Has severe headache, pain in the back, and feels very weak in his limbs. The spleen can be felt, but there is no tenderness. Pulse 111, quick and small; tongue yellow and a little moist.

Prescription. ℞. Quiniæ sulphatis, ʒj.

Acid sulphuric aromat., gtt. xxx.

Aquæ, f℥vj. M.

S. A tablespoonful to be taken every hour.

This case progressed very much in the same way as case II, excepting that the pains he at first complained of grew less severe and disappeared altogether about the eighth day, when he had most fever, and his pulse was 122. He continued with but little alteration for three or four days. The spleen could no longer be felt, (Nov. 21st.) He experienced the effect of the quinine on his head without any increase of dose; after which it was lessened, as in the preceding case, and now, Nov. 25th, convalescence seems established, and he is directed to take it three times a day.

Case IV.—January 5th. I was sent for this morning to the daughter of L. H., æt. 5. She has been troubled with diarrhœa for a week, until the last two days, during which she has had only one alvine evacuation. Has complained of pain in the bowels, particularly in the left side, and of being cold. Has no appetite; thirst is increased; has a dry hacking cough, and is very restless. She now lies perfectly quiet; is in partial perspiration; has no disposition to be interfered with. The eyes are inflamed; tongue is white, a little moist. Pulse 132, very quick and small. Does not complain of any pain.

Prescription. ℞. Ol. ricini.

Syr. acaciæ, *aa*, ʒj. M.

Diet. Toast water or gum water.

6th. Has been more restless; sick at the stomach; more thirsty. The tongue is drier; pulse 138; there is still partial perspiration; medicine has not operated; and, ℞. Ol. ricini, ʒj.; syr. rhei, ʒij. M.—is prescribed.

7th. Medicine has acted four times. Sickness at the stomach is less. She was very restless during the night. Has epistaxis; increased thirst; the skin is hot and dry; pulse 146, very quick and small; tongue more yellow and dry; snuffing respiration.

Prescription. ℞. Quiniæ sulphatis, gr. xvj.
Acid. sulphuric aromat., gtt. x.
Aquæ, ℥ij. M.

S. A teaspoonful to be given every hour.

8th. Still very restless. Has had no evacuation from the bowels; has more sickness at the stomach, rejects the medicine; increased heat and dryness of the skin; pulse is 150; the mouth is very dry, and the tongue darker coloured.

Prescription. ℞. Quiniæ sulphatis, gr. xvj.

Liq. morphiæ sulphatis, f℥ss.

Acid. sulphuric aromat., gtt. xx.

Aquæ, ℥jss. M.

S. A teaspoonful to be given every hour.

9th. Delirious all the first part of last night. Has had a very free and fœtid evacuation from the bowels. Epistaxis. There are sordes about the teeth and lips; the tongue is black and cracked. She seems very thirsty when drink is offered, but does not ask for anything. Pulse 134. The skin is very hot and dry. She picks at her mouth and nose a great deal.

10th and 11th. The same prescription was continued, and she is now (12th) more delirious. The pulse is 158; the skin exceedingly hot and dry; the tongue bleeds; the nose is stopped with hardened blood. Sudamina. Almost constant motion of the hands; picking the mouth and nose, or bed clothes. Slides down in bed, &c.

Prescription. A teaspoonful of castor oil.

℞. Quiniæ sulphatis, ʒss.

Liq. morphiæ sulphatis, ℥ss.

Acid. sulphuric aromat., gtt. xx.

Aquæ, ℥jss. M.

S. A teasaoonful to be given every hour.

17th. Up to this time she has continued with the same serious symptoms she manifested on the 12th. The castor oil operated three times, and she has had three or four alvine evacuations daily since. She now picks at her ears, shrugs her shoulders, and shows that she experiences some unnatural sensation about the ears. Pulse 130, not so quick. The tongue is very black, cracked and rough. Prescription same as on the 8th.

18th. She has had seven alvine evacuations, the last not so dark coloured nor so fetid. Has been less delirious; does not take her drinks so eagerly; says there is something in her ears. Pulse 120; mouth a very little moist; skin not so hot. The same ℞. is to be continued every two hours.

19th. The symptoms are still more favourable. Has had three alvine evacuations, appearances still better. Delirium all gone; skin more natural; sordes cleaning about her mouth, which is moist, and the edges of the tongue are beginning to clean. Pulse 108. Same prescription continued.

24th. She has continued to improve under the same treatment, and is now decidedly convalescent. The quinine is ordered every six hours.

Cases fourth and fifth, of which I also have notes, are not sufficiently unlike the second and third to need publication; they were managed in the same way, and the result was equally successful.

REMARKS.

When once typhoid fever has become established in the system, I believe it is generally admitted that it is very difficult, if not altogether impracticable, to cut it short by any means. The efforts of the practitioner are consequently restricted to tempering the morbid actions, and especially to the removal of any hyperæmia that may supervene. That such is the correct view, when contrasted with such agents as are generally used to break up fever, it is presumed no intelligent physician will contradict; for to use violent remedies, of the caustic or cathartic kind, in any stage of this disease, appears to me would show such ignorance of its pathology as the present facilities for studying that branch would utterly condemn.

So far as I have used the sulphate of quinine, I have not been led to think that the duration of the disease has been in the least affected by it; but as soon as other opportunities shall occur, I shall not hesitate to give it a thorough trial at the commencement, as my use of it has satisfied me that if it does not succeed in arresting the disease, there need be no fears of its increasing the intestinal affection, which has thus far caused me to be extremely cautious in administering the quinine and to closely watch its effects.

Since fully experiencing the effects of sulphate of quinine in my own person, and closely observing them in others, I have not in the least regarded any of the directions or opinions of individuals who suppose it to have a pernicious influence when given in the time of fever. In Northampton county, Virginia, during the autumn of 1842, I had greater opportunities, and I think I heeded them, of testing its effects when given in the hot stage of remittent fever than is usually afforded; during which time, and the autumn of 1843, I successfully so used it in over four hundred cases, almost to the entire exclusion of mercury in any of its forms, not even combined with an emetic at the commencement; and in my opinion, (and my experience in the use of it fully establishes it to my own mind,) it is a better preventive of congestion, which is the only danger in those fevers, than mercury or anything else with which I am acquainted.

Here I will take the liberty to digress, in order to notice, with much respect, the opinion of my friend Dr. Upshur, of Norfolk, on "Miasmatic Fevers," as published in a late number of the Examiner. In his notice of the unusual prevalence of fevers, and what he attributes to the treatment of them in Northampton, in the autumn of 1842, I think, upon further inquiry, he will perceive that he has confined his opinion within too narrow bounds, when he thinks that the great mortality during winter there is to be attributed to the almost exclusive use of mercurials in the management of the autumnal fevers. Now, previous to 1842, I entertained a similar opinion, but my own practice there led me to give it a more extensive scope. As remarked above, I scarcely used mercury, in any form, in a single case, for it oftentimes happens in those fevers, that the length of time occupied in getting a patient under the influence of mercury, would cure him under a much more efficacious and milder course of treatment. I usually commenced my treatment with an emetic of ipecacuanha and tartar emetic combined, which I followedby a mild cathartic of castor oil, or rhubarb and magnesia if necessary, and then commenced immediately giving the sulphate of quinine, and have many times repeated it hourly, in doses varying from five to thirty grains, until, after getting the patient under the influence of it, in doses frequent enough and large enough to keep up its influence until the time for the cold stage had passed, which it always will if the patient is fully under its influence. I have found, too, that so far from causing additional excitement in such cases, it is just the reverse when its operation is favorable—and I have never seen a case of remittent or intermittent fever when it was otherwise,—occasioning a diminution of the frequency of the pulse, (as first noticed, I believe, by Dr. Thomas Fearn, of Huntsville, Alabama;) and so far from doing harm in fever, independent of the expected cold stage, it is actually beneficial.

If in such doses it disagrees with the stomach, as it sometimes does, I usually add a small proportion of the sulphate or acetate of morphia to it, which has not only the effect of a corrective, but adds to its efficacy.

Bloodletting is one of the best remedies for the obstinate sick stomach, so apt to occur in some of those fevers, I have ever used; and for the removal of congestion, after it has once commenced, which is not a frequent event, if the sulphate of quinine is early and boldly given, it is almost indispensable.

Such is an outline of my method of practice in the treatment of the remittent fevers of Northampton; and as I never lost a case of simple intermittent or remittent fever, I was under no necessity of resorting to mercurials or any other treatment. But my patients shared as largely in the mortality prevalent there during the following winter, as those of some of my brethren who so rigidly adhere to the mercurial practice. From what observations I was able to make, it was not by any means exclusively in consequence of the treatment which had been previously pursued. So far only as the mercurial treatment added to the debility of those who had been ill, it rendered them more subject to congestive fever at the coming winter; whilst a protracted convalescence from any other cause, or where they had an attack of fever late in the autumn, and were consequently much enfeebled, anæmic, &c. at the commencement of winter, they were usually unable to resist sudden congestion of different organs, which is so very apt to follow the slightest exposure. And, according to my experience, the cases which were most quickly fatal, were those which had been affected with fever latest in the season, or were most debilitated at the onset of the congestive attack; or in other words, those who were least able to bear it, on account of sufficient time not having elapsed for the energies of the system to be fully restored, were most apt to have typhoid affections of the lungs, brain, andchylopoietic viscera, and least able to resist them.

I think that the reason the winters in Norfolk are comparatively free from inflammations of a typhoid character, is not because the physicians there rely more upon the use of quinine than mercurials, but because remittent fever is not near so prevalent in that locality as it is in Northampton; and if it were so, such attacks would not be so common, because the situation of Norfolk renders it free from those piercing winds which commence with the winter in Northampton.

In my use of the sulphate of quinine in typhoid fever, I have noticed in every case a reduction in the frequency of the pulse as soon as the system began to be influenced by it, which is what I have been unable to effect by any other remedy, without other effects much more deleterious than tinitus aurium; and whatever its toxical effects may be on animals, I have never noticed any on the human subject. I have taken a drachm of it myself without experiencing any other unpleasant sensation than a peculiar roaring in the ears, and an indistinctness of common sounds. I have seen pregnant and nursing females, who are much more easily brought under its influence than others,* so much affected by it as to be unable to distinguish sounds, and to liken the sensation which it excites in the head to that produced by turning swiftly round. These effects rarely last for a

* I think this susceptibility to the impression of the quinine is owing to the irritability of the nervous system in these conditions, as I have observed that other individuals who are noted as being peculiarly "nervous," are also more easily brought under the influence of it.

longer period than twenty-four hours, if the remedy be discontinued.

I have kept up the impression of the quinine in a slight degree, in typhoid fever, for more than a week, and from the six cases in which I have successfully used it, I am inclined to believe that if the system can be brought under its influence before hyperæmia has actually begun in any of the internal organs, and be kept so until the symptoms indicate that there is no longer danger of it, the most dangerous complications and sequelæ may be avoided. In no case in which I have used it has it been necessary to employ wine or other stimulants, and at the termination of the disease the system was much less prostrated, and much better prepared for what I deem a very decided advantage—a short convalescence.

I am well satisfied that the condition in which the six cases, taken successively, were left at the termination of the disease, was not in consequence of their being mild cases, any further than as they were affected by the treatment, if the symptoms at the beginning of the disease are any indication of what may be expected of its later character. In every case, just as soon as the patient was under the influence of the quinine, much greater regularity was observed in the progress of the disease, and there was no tendency whatever to that troublesome complication, inflammation and enlargement of the spleen, which is usually followed by others still more severe and alarming—as diarrhœa, cold sweats, cold extremities, purple cheeks, involuntary discharges, &c., which call for the most active stimulants to sustain vital action, at the expense frequently of its proving fatal the sooner, in consequence of their aggravation of the inflammation of the follicles of the intestines and mucous membranes. From what I have already seen of its effects, I am led to anticipate much more from it, if it should be thought worthy of trial by more experienced and acute observers than myself.

Periodicity in disease is not always so manifest as to be at once evident; but that there is as much evidence of periodicity in typhoid fever as in many other affections equally curable by sulphate of quinine, is beyond question. As remarked by Prof. T. D. Mitchell, "we cure an ague and fever by sulphate of quinine, no matter whether it be a true and open ague or a masked intermittent. And we cure many cases of genuine neuralgia, unattended by chill or fever at all perceptible, by the same agent. These are common cases, with which all practitioners are familiar, and they clearly set forth the adaptation of the remedy on the ground of intermittence." But whether typhoid fever possesses the same common property is not yet sufficiently demonstrated to place it beyond doubt; and its curability by sulphate of quinine no more establishes it, than does the circumstance of syphilis giving way and being cured by mercury make it a symptom in its diagnosis.

*Waterbury*, Ct., Feb. 16th, 1846.

